# Editorial: Dance and Aging

**DOI:** 10.3389/fspor.2022.908688

**Published:** 2022-04-26

**Authors:** Pirkko Markula, Marianne Clark, Donna Goodwin

**Affiliations:** ^1^Faculty of Kinesiology, Sport, and Recreation, University of Alberta, Edmonton, AB, Canada; ^2^The Vitalities Lab, Centre for Social Research in Health and the Social Policy Research Centre, University of New South Wales, Kensington, NSW, Australia

**Keywords:** aging, dance, dance benefits, dance experience, enjoyment of dance

The world's population is aging. The United Nations et al. (2019) estimate that in 2019, there were 703 million older persons aged 65 and older and this number is expected reach over 1.5. billion in 2050. The populations are currently significantly older in Australia and New Zealand, Europe, and North America than the other parts of the world. Nevertheless, the quality of life of the aging population has become an increasing concern globally.

One essential aspect for positive aging is physical activity. According to recent research (e.g., Raafs et al., 2020), moderate physical activity that combines physical, mental, and social demands is the most advantageous for enhancing older people's quality of life. In their meta-analysis and systematic review, for example, Roberts et al. (2017) concluded that high multitask, social activities such as dance, improved physical, mental, and social abilities of mature individuals. Yet the majority of older people, particularly women, do not meet the recommended amount of daily physical activity to sustain wellbeing. It is important, therefore, to consider what type of physical activity may inspire older adults to become more active.

With the image of a professional dancer as physically fit, hyperflexible, and superbly coordinated, dance can appear unsuitable for mature individuals. In addition to professional dance, however, there are multiple dance forms that can attract different types of participants. As a multitask activity with a strong social component, dance, thus, can have the capacity to attract both mature women and men to physical activity. In addition to improving both cognitive and sensorimotor performance, dance incorporates interesting and enjoyable ways to improve mental, social, and emotional wellbeing. While dance has the potential to enrich every area of life contributing to health and better functioning of mature persons, currently, many researchers examining the benefits of dance have focused on measuring isolated variables such as balance, motivation, memory, or self-expression.

While several scholars of aging and physical activity have used scientific evidence to demonstrate that dance is beneficial to the health and wellbeing of older people, this type research can also separate physical and emotional benefits from their social contexts. While health outcomes are important, it is also crucial to consider how the aging body (and mind), when socially constructed as necessarily in decline, can be considered only as a burden to society. Therefore, it is essential to highlight how aesthetic, cultural, and social meanings enhance mature dancers' lives. While some social scientists have investigated individual experiences of dancing at mature age, this research often appears in different publications from the bio-medical and neuroscientific studies of dance benefits. Consequently, the dialogue and collaborative engagement with different research traditions is often missing.

The goal of this Research Topic was to gather together research that addresses the multiple functions of dance on the quality of life of older people and/or critically analyzes the interrelated connections of physical, psychological, aesthetic, cultural, and social meanings of dancing for its participants. As a result, the collection of articles in this Topic highlights the multifaceted meanings and functions of aging and different dance forms in the contemporary society.

The authors show how dance, ranging from professional contemporary dance, recreational ballet, social dance, or improvisation dance classes, can enhance older people's quality of life, but also challenge ageism in society. They examine, using both quantitative and qualitative methodologies, how mature adults engage, experience, and enjoy their dance activities. For example, when Thumuluri et al. piloted an intervention trial using IMPROVment^®^, an improvisation dance class, to quantitively test its effect on early-stage dementia, they found increases in quality of life and balance. Using a qualitative approach in their Latourian inspired study “Women's Articulations of Aging,” Jeffrey et al. interviewed recreation ballet dancers to find that when dancing, the participating women were given an opportunity to think differently about their bodies. In their interactionist sociological study, “Dancing My Age,” Heikkinen and Wilinska discovered that the interviewed social dancers' emotional energy overrode concerns of aging when on the dance floor.

Some of the authors are mature dancer-scholars themselves and used their multiple conceptual toolkits to reveal ageism in dance culture and change the image of the western theater dance only as a young people's affair. For example, Nikolai recounted her dance making process in her article “Move: Durational Dance Making, as We Age” and Markula et al. analyzed how their dance group *Initial 6* challenged ageism in contemporary dance in their memory-work study, “It Can Be Magic.” Bustad and Engelsrud's document analysis of previous dance research framed these experiences in a larger context of why “everybody, except aging professional dancers can dance.”

In this research dialogue, dance and aging intertwine to present experiences of complex physical, emotional, and social engagement with both enjoyment and doubt of one's physical ability, intense involvement in the special “magic of dance,” and inclusion with a special group of like-minded, yet diverse people. As such, the works on this Research Topic reveal dance as a positive space for mature individuals to be physically active. Engaging dancers with different abilities who practice various dance styles in Europe, Oceania, and North America, the Research Topic aims to encourage and initiate further examinations of other geographical areas, dance forms, dancing identities, and positive experiences of aging.

## Author Contributions

PM wrote the draft that was commented on and edited by DG and MC. All authors contributed to the article and approved the submitted version.

## Conflict of Interest

The authors declare that the research was conducted in the absence of any commercial or financial relationships that could be construed as a potential conflict of interest.

## Publisher's Note

All claims expressed in this article are solely those of the authors and do not necessarily represent those of their affiliated organizations, or those of the publisher, the editors and the reviewers. Any product that may be evaluated in this article, or claim that may be made by its manufacturer, is not guaranteed or endorsed by the publisher.
